# Clinical evaluation of bispectral index‐guided closed‐loop infusion of propofol for preschool children: A multi‐center randomized controlled study

**DOI:** 10.1002/ped4.12449

**Published:** 2024-08-27

**Authors:** Lei Hua, Bin Du, Yunxia Zuo, Huacheng Liu, Jianmin Zhang

**Affiliations:** ^1^ Department of Anesthesiology Beijing Children's Hospital Capital Medical University, National Center for Children's Health Beijing China; ^2^ Department of Anesthesiology West China Hospital Sichuan University Chengdu Sichuan China; ^3^ Department of Anesthesiology and Perioperative Medicine The Second Affiliated Hospital and Yuying Children's Hospital of Wenzhou Medical University Wenzhou Zhejiang China; ^4^ Key Laboratory of Pediatric Anesthesiology Ministry of Education Wenzhou Medical University Wenzhou Zhejiang China; ^5^ Key Laboratory of Anesthesiology of Zhejiang Province Wenzhou Medical University Wenzhou Zhejiang China

**Keywords:** Closed‐loop, Target‐controlled infusion, Bispectral index, Preschool children

## Abstract

**Importance:**

The closed‐loop infusion system can automatically adjust and maintain the depth of anesthesia by using the propofol target‐controlled infusion (TCI) model under the feedback guidance of the bispectral index (BIS).

**Objective:**

To evaluate the safety and superiority of closed‐loop TCI of propofol guided by BIS during maintenance of generalized intravenous anesthesia for preschool children.

**Methods:**

A total of 120 children aged 1–6 years were enrolled and were divided into a closed‐loop feedback group (Group C) and an open‐loop manual control group (Group O), with 60 participants in each group. For anesthesia maintenance, the propofol infusion rate was adjusted by the injection system under the guidance of BIS in Group C and was manually adjusted by anesthesiologists according to the BIS and clinical experience in Group O, to maintain a BIS level of 50. The time ratio of adequate anesthesia (40 ≤ BIS ≤ 60), light anesthesia (BIS > 60), and deep anesthesia (BIS < 40) were recorded.

**Results:**

A total of 119 patients (59 in Group C and 60 in Group O) were enrolled in the study. Group C demonstrated a higher time ratio of adequate anesthesia (*P* = 0.014) compared to Group O. The time ratio of light anesthesia and the global score was lower in Group C than in Group O (*P =* 0.010, *P* = 0.015, respectively). The frequency of adjustment per unit of time was higher in Group C for propofol (*P* < 0.001), while it was lower for remifentanil (*P* = 0.010).

**Interpretation:**

BIS‐guided closed‐loop infusion of propofol is safe and effective for preschool children. The depth of anesthesia is controlled more accurately and smoothly.

## Graphical abstract

The closed‐loop infusion system feeds back the monitoring output signal to the control module to automatically achieve and maintain the target goal. Through a multi‐center study, we compared the closed‐loop feedback and open‐loop control of propofol under the guidance of bispectral index and verified that the closed‐loop propofol infusion system can be used safely and effectively for anesthesia in preschool children

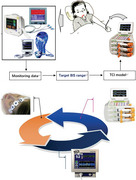

## INTRODUCTION

Closed‐loop infusion systems use computer technology and reliable pharmacological effect measurements to automatically achieve and maintain the preset target of injection systems by feeding back the output signals of the monitoring module to the control module.^1^ Although several target‐controlled infusion (TCI) models of propofol and remifentanil are suitable for preschool‐aged children, who are in a stage of rapid growth and development,^2^ their pharmacokinetic models cannot fully address the individual differences in sedation and analgesia. Studies of adults^3^, ^4^, ^5^ have confirmed that the closed‐loop TCI of propofol guided by the electroencephalogram bispectral index (BIS) is superior to manual control, with a higher proportion of time spent in the BIS = 40–60 range, indicating a more accurate and stable depth of anesthesia. Few studies have focused on children, especially preschool‐aged children. Therefore, this study aimed to examine the safety and superiority of BIS‐guided closed‐loop TCI of propofol in preschool‐aged children.

## METHODS

### Ethical approval

The study was approved by the ethics committee of Beijing Children's Hospital, Capital Medical University (2016‐Y‐020‐B). Each subject and guardian were informed of the study details during the preoperative visit and signed informed consent for the study.

### Study design

This was a multicenter prospective randomized controlled study. There were three research subcenters (Beijing Children's Hospital, Capital Medical University; West China Hospital of Sichuan University; and the Second Affiliated Hospital of Wenzhou Medical University). The inclusion criteria were as follows: 1–6 years old; American Society of Anesthesiologists (ASA) level I–II; a plan to undergo elective noncardiac and noncranial surgery under general anesthesia; a body mass index not exceeding 20% above the normal range; and an expected operative time more than 1 h. The exclusion criteria were concomitant central or peripheral nervous system disease; liver or kidney dysfunction or coagulation function abnormalities of more than 2 times the normal level; and intraoperative blood dilution or hypothermia.

The subjects were randomly divided into two groups using a random number table: the closed‐loop feedback group (Group C) and the open‐loop manual group (Group O), at a ratio of 1:1.

### Anesthesia procedure

After entering the room, the patients in both groups were given 0.05 mg/kg midazolam intravenously and were continuously monitored. The monitored vital signs were electrocardiograph, oxygen saturation (SpO_2_), noninvasive blood pressure, BIS, and train of four (TOF). The induction phase of the two groups was defined as the period from the start of the infusion of propofol (Jing'an, Fresenius) and remifentanil (Ruijie, Humanwell Healthcare) until the BIS was less than 60 for 30 s. The maintenance phase was defined as the period from the above time point to the end of the infusion of propofol and remifentanil. The Paedfusor model was adopted for the TCI of propofol, and the Minto model was used for the TCI of remifentanil. The infusion pump used the total venous three‐way monitoring automatic injection system (CONCERT‐CL, Guangxi Veryark). For the closed‐loop feedback channel of propofol, based on the average BIS from sampling every 5 seconds for 3 minutes, corresponding to the target concentration of propofol, according to the fluctuating difference between the actual BIS and the preset target BIS, the system automatically adjusted the target concentration by changing the propofol infusion rate.

During the induction phase, based on experience, the anesthetist set the initial plasma target concentration of propofol at 3–5 µg/mL and the initial plasma target concentration of remifentanil at 2–6 µg/mL. After the eyelash reflex disappeared and the BIS was < 60, both groups were given rocuronium bromide 0.6 mg/kg. After the TOF reached 0 and muscle relaxation was complete, the endotracheal tube or laryngeal mask was placed for mechanical ventilation. In the maintenance phase, total intravenous anesthesia (TIVA) was given in both groups. In Group C, the infusion system automatically adjusted the plasma target concentration of propofol TCI based on the actual change in the BIS. In Group O, the anesthesiologist, based on their clinical routine and experience and with reference to the BIS monitoring value, manually adjusted the plasma target concentration of the propofol TCI so that the BIS was kept as stable as possible near the target value of 50 in both groups. The TCI concentration of remifentanil in the two groups was adjusted by the anesthesiologist based on the vital signs and surgical progress of the patients to keep it at 2–6 µg/mL. Rocuronium was manually added to the two groups based on the TOF value monitored by the C channel, and the use of rocuronium was stopped 30 minutes before the end of the surgery. Twenty minutes before the end of the surgery, both groups received a slow intravenous bolus injection of 0.1–0.5 µg/kg sufentanil for postoperative pain. All drug infusions were expected to be stopped 5–10 min before the end of surgery.

During the emergence phase, the patients resumed spontaneous breathing, with SpO_2_ > 95%, responsiveness, and a T4/T1 score ≥ 90%. The tracheal tube or laryngeal mask was removed after the vital signs were stable. All patients in both groups were transferred to the post‐anesthesia care unit (PACU) after surgery. The transfer criteria were that the patient was awake, SpO_2_ ≥ 95% on room air, and had no acute anesthesia or surgical complications.

### Observation indicators

The main observation indicator of this study was the ratio of the time of adequate anesthesia (40 ≤ BIS ≤ 60) in the maintenance phase to the total time in the maintenance phase, that is, the adequate anesthesia time ratio. Secondary observation indicators included the time ratio of light anesthesia (BIS > 60) and deep anesthesia (BIS < 40) in the maintenance phase and the global score (GS) of the infusion device; the differences between the two groups using propofol and remifentanil in terms of the dosage per unit time, TCI plasma concentration and number of adjustments; and the time to extubation, time to wakefulness, and PACU stay time in the two groups.

The GS was affected by the adequate anesthesia time ratio, the median absolute performance error (MDAPE), and the wobble, which reflected the overall performance of the closed‐loop infusion system. GS = (MDAPE + wobble)/% (when 40 ≤ BIS ≤ 60). When MDAPE and wobble were lower, and at the same time the proportion of adequate anesthesia time was high, the GS score was low, indicating good performance of the closed‐loop system.^6^

### Sample size calculation

According to the results of previous studies in the literature,^4^ the ratio of full anesthesia time (BIS 40–60 time/total time) of open loop TCI system was 70% ± 19%. This study assumed that the closed‐loop TCI system could improve the ratio of full anesthesia BIS 40–60 times by 20%. Set the test efficacy 1−β as 80%, the two‐sided test Class I error probability α as 5%, the loss rate as 20%, and the non‐inferiority test. The total sample size was 120 cases, with 60 cases in each group, which was completed by the cooperation of three research centers, with a total of 40 cases in each center.

### Statistical analysis

SA9.4 software was used for calculations. Statistical tests were performed using two‐sided tests. The test level α was 0.05, and *P* < 0.05 was considered statistically significant. Quantitative data are expressed as mean ± standard deviation or median (interquartile range), and qualitative data are presented as proportions or percentages. The quantitative data were subjected to normality and homogeneity of variance tests followed by the *t‐*test, *t’*‐test, and Wilcoxon rank sum test. The qualitative data were analyzed using the chi‐squared test, corrected chi‐squared test, and Fisher's exact probability method to compare differences between groups.

## RESULTS

In this study, 120 subjects were randomly enrolled from three subcenters, with 60 subjects each in Group C and 60 subjects in Group O. In Group C, one patient was excluded from the statistical analysis because the actual operative time was less than half an hour, which was significantly shorter than expected. The demographic data and baseline characteristics of the two groups were similar (Table 1).

**TABLE 1:** Baseline characteristics of two groups

Group	Age	Gender	Height	Weight	ASA	Operating time
	(year)	(M/F)	(cm)	(kg)	(I/II)	(min)
Group C (*n* = 59)	2.42 ± 1.49	43/16	92.37 ± 12.45	14.14 ± 4.36	37/22	93.00 (64.00–130.00)
Group O (*n* = 60)	2.43 ± 1.49	47/13	92.53 ± 12.93	13.61 ± 3.60	35/25	85.85 (63.50–138.75)
*χ* ^2^ */t/Z*	0.086	0.480	−0.053	0.644	0.239	0.320
*P*	0.932	0.489	0.958	0.520	0.625	0.750

Data are shown as *n* or mean ± standard deviation or median (interquartile range).

Abbreviations: ASA, American Society of Anesthesiologists; F, female; Group C, closed‐loop feedback group; Group O, open‐loop manual control group; M, male.

The primary outcome indicator of the study, the adequate anesthesia time ratio in the maintenance phase (40 ≤ BIS ≤ 60), was 79.92% ± 14.09% for Group C and 73.62% ± 16.33% for Group O (*P* = 0.014). In the maintenance phase, the ratio of the duration of light anesthesia (BIS > 60) was significantly lower in Group C than in Group O [5.30% (2.20%–11.90%) vs. 10.05% (3.93%–16.58%), *P* = 0.010]. There were no significant differences in the deep anesthesia time ratio (BIS < 40) or the mean BIS between groups. The GS of Group C was lower than that of Group O [(23.27 (17.34–33.95) vs. 28.56 (22.03–37.03)], *P* = 0.015] (Table 2).

**TABLE 2:** Maintenance phase effectiveness analysis

Group	Time ratio of 40 ≤ BIS ≤ 60 (%)	Time ratio of BIS < 40 (%)	Time ratio of BIS > 60 (%)	BIS mean value	GS
Group C (*n* = 59)	79.92 ± 14.09	9.10 (5.00–15.77)	5.30 (2.20–11.90)	48.19 ± 2.95	23.27 (17.34–33.95)
Group O (*n* = 60)	73.62 ± 16.33	9.50 (3.50–19.03)	10.05 (3.93–16.58)	49.70 ± 4.43	28.56 (22.03–37.03)
*t/Z*	2.448	0.066	−2.586	−1.597	−2.442
*P*	0.014	0.947	0.010	0.110	0.015

Data are shown as mean ± standard deviation or median (interquartile range).

Abbreviations: BIS, bispectral index; Group C, closed‐loop feedback group; Group O, open‐loop manual control group; GS, global score.

During the maintenance phase, the dosage per unit of time and the mean target concentrations of propofol and remifentanil were similar between the two groups. The number of adjustments of propofol per unit time in Group C was significantly greater than that in Group O [21.37 (17.18–27.93) vs. 4.94 (2.97–6.99), *P* < 0.001], and the number of adjustments of remifentanil in Group C was significantly less than that in Group O [0.85 (0.43–2.22) vs. 1.66 (0.83–2.77), *P* = 0.010] (Table 3). No significant difference was observed in the time to extubation, time to wakefulness, or PACU stay time between the two groups (Table 4).

**TABLE 3:** Comparison of drug use between the two groups

	Propofol			Remifentanil		
	Average dosage	Mean target concentration	Number of adjustments	Average dosage	Mean target concentration	Number of adjustments
Group	(mg·kg^−1^·h^−1^)	(µg/mL)	(times/h)	(mg·kg^−1^·h^−1^)	(µg/mL)	(times/h)
Group C (*n* = 59)	13.61 ± 2.84	4.10 ± 0.72	21.37 (17.18–27.93)	32.87 ± 12.41	2.99 ± 0.91	0.85 (0.43–2.22)
Group O (*n* = 60)	12.90 ± 2.98	3.95 ± 0.76	4.94 (2.97–6.99)	32.36 ± 9.68	2.89 ± 0.69	1.66 (0.83–2.77)
*t/Z*	1.106	0.892	8.839	−0.338	0.352	−2.710
*P*	0.269	0.373	<0.001	0.736	0.725	0.010

Data are shown as mean ± standard deviation or median (interquartile range).

Abbreviations: Group C, closed‐loop feedback group; Group O, open‐loop manual control group.

**TABLE 4:** Comparison of extubation time, waking time, and post‐anesthesia care unit (PACU) residence time between the two groups

	Extubation time	Recovery time	PACU residence time
Group	(min)	(min)	(min)
Group C (*n* = 59)	20.78 ± 9.51	46.80 ± 17.45	39.78 ± 13.62
Group O (*n* = 60)	19.85 ± 6.55	45.22 ± 20.23	39.85 ± 14.89
*t*	−0.285	0.524	1.236
*P*	0.776	0.601	0.236

Data are shown as mean ± standard.

Abbreviations: Group C, closed‐loop feedback group; Group O, open‐loop manual control group; PACU, post‐anesthesia care unit.

## DISCUSSION

The depth of anesthesia is an important concern of anesthetists during general anesthesia. Too‐deep anesthesia can lead to circulatory depression, delayed postoperative emergence, and increased postoperative complication and morbidity, while too‐light anesthesia can lead to intraoperative awareness and severe hemodynamic fluctuations.^7^ Maintaining an appropriate depth of anesthesia is critical for improving the quality of anesthesia, promoting the rapid recovery of patients, and reducing the incidence of anesthesia complications.^8^

For a long time, the BIS has been an important monitoring indicator reflecting the depth of anesthesia. The BIS can be applied not only to adults but also to children or even infants.^9^ Struys et al.^10^ noted that the use of the BIS as a controlled variable combined with the model‐based closed‐loop system of propofol administration is clinically acceptable. Therefore, the BIS is a good feedback indicator for closed‐loop propofol infusion, and by setting the target range and system feedback regulation, we expect to achieve the ideal depth of anesthesia.

Closed‐loop feedback infusion uses specific pharmacokinetic and pharmacodynamic models to develop systematic dosing regimens using computer programs, and the depth of anesthesia and muscle relaxation are monitored as feedback indicators to programmatically adjust infusion parameters so that monitoring indicators are controlled within predefined ideal ranges.^11^ The TCI infusion models of propofol and remifentanil selected in this study were Paedfusor and Minto, respectively. Previous studies^12^, ^13^ have indicated that the above models are suitable for application to the age group of children in the present study. Using this infusion model, the mean target concentrations of propofol and remifentanil in the two groups were roughly equivalent.

Large randomized controlled studies in adults^4^, ^14^ have shown that the ideal BIS control time, system GS, and analgesic effect are significantly better in the closed‐loop group than in the manual group. A meta‐analysis that included 12 clinical studies^15^ pointed out that, compared with manual control, the closed‐loop system of BIS‐guided total intravenous anesthesia used less propofol during the induction phase, better maintained the target depth of anesthesia, and led to a shorter recovery time. In the present study, the patients of both groups stayed at an appropriate depth of anesthesia. Group C had a greater adequate anesthesia time ratio than Group O. This shows that closed‐loop systems can provide a more sensitive and rapid feedback regulation of the anesthetic state by identifying and predicting changes in the BIS, thereby enabling the patient's depth of anesthesia to achieve a better steady state. The duration of light anesthesia in the maintenance phase in Group C was significantly shorter than that in Group O, indicating that the closed‐loop TCI system may be more conducive to preventing intraoperative awareness in patients receiving total intravenous anesthesia. The GS of Group C was significantly lower than that of Group O, indicating that the internal performance of the closed‐loop infusion system was stable and advantageous.

Group C needed 21.37 (17.18–27.93) propofol adjustments per hour, which was far greater than that in Group O. Such frequent feedback adjustments are almost impossible to achieve manually in an open‐loop system, indicating that the feedback system adjustment has a high degree of quick responsiveness, which greatly reduces the workload of the anesthesiologists and allows them to focus more on the surgical process and systemic changes in patients.^16^ Similarly, Dussaussoy et al.^17^ showed that during the induction and maintenance of anesthesia, the anesthesiologists in their propofol closed‐loop group observed the monitors and infusion system less often than the manual adjustment group, while the results of adequate anesthesia time and medication were similar between the two groups, indicating that the closed‐loop system frees anesthesiologists from the frequent work of manual intervention and adjustment. In the present study, Group C had fewer adjustments of remifentanil per unit time than Group O. This may suggest that with closed‐loop infusion, the hemodynamics of pediatric patients are more stable, so there is less need to adjust the infusion level of analgesic drugs.

We saw no significant difference between the two groups in the time to extubation, time to wakefulness, or PACU stay time. This indicates that both closed‐loop infusion systems and open‐loop manual adjustment are equally safe and reliable in clinical application, with no significant differences in their impact on the postoperative recovery phase, and that closed‐loop feedback does not increase the risk of delayed extubation or awakening in pediatric patients.

In a closed‐loop target‐controlled study of children, Orliaguet et al.^18^ enrolled 42 pediatric patients aged 7 to 14 and compared the effect of closed‐loop TCI and manual adjustment of propofol on the depth of anesthesia. Their closed‐loop group spent more time at a BIS of 40–60, demonstrating that the BIS‐guided closed‐loop control system can be used successfully in clinical practice and is superior to the manual control system. Biswas et al.^19^ studied a total of 40 children with ASA II–III undergoing extracorporeal circulation cardiac surgery, who were randomly divided into a closed‐loop infusion of propofol group and a traditional manual control group. The results showed similar target BIS ranges (50 ± 10) and hemodynamic stability in both groups, and the amount of propofol and phenylephrine used in the closed‐loop group was lower, indicating that closed‐loop propofol anesthesia is equally applicable in challenging cardiac surgery. The present study was designed as a multicenter randomized controlled clinical trial, focusing on a special population of children, with a large sample size. It provides high‐quality evidence supporting the application of a closed‐loop target control system in preschool‐aged children and demonstrates a certain level of innovation.

Based on the wavelet analysis of feedback indicators, West et al.^20^ attempted to add closed‐loop control of the second drug, remifentanil, to improve the stability of the closed‐loop feedback control system in the presence of variable surgical stimuli. Their study of 127 patients showed that adding closed‐loop control of remifentanil improved the overall control performance and optimized the control of both drugs using a single sensor. Nagata et al.^21^ employed a new system algorithm for the closed‐loop administration of propofol, remifentanil, and rocuronium. Compared with those in the manual control group, the percentage of time with adequate control of sedation, analgesia, and muscle relaxation in the closed‐loop group was 87.21% ± 12.79%, which was not lower than the 65.19% ± 20.16% in the manual group (*P* < 0.001), and the closed‐loop group had fewer incidents than the manual group.

This study has some limitations. In a study of closed‐loop TCI of propofol in children, Hu et al.^22^ carried out a logistic regression analysis of the factors influencing the ratio of adequate anesthesia and showed that young age was one of the independent risk factors for reducing the adequate anesthesia time ratio, with an age cutoff value of 17 months, indicating that younger children should be more closely observed and controlled. The subjects of the present study were 1–6 years old, and their ages were not further stratified for age‐group comparisons. The reliability of the closed‐loop TCI device (CONCERT‐CL, Guangxi Veryark) has been verified in previous studies targeting adults.^4^ In the present study, we only verified the closed‐loop feedback effect of a single propofol channel, and we only monitored muscle relaxation without closed‐loop drug administration. Therefore, its clinical applicability was not verified.

In summary, BIS‐guided closed‐loop infusion of propofol is safe and effective for preschool‐aged children, as it maintains the depth of anesthesia more accurately and stably than manual adjustment.

## CONFLICT OF INTEREST

The authors declare no conflicts of interest.
